# Di-*n*-butyl 5-amino­isophthalate

**DOI:** 10.1107/S1600536809024696

**Published:** 2009-07-04

**Authors:** Tian Zhou, Ru-Jin Zhou, Zhe An

**Affiliations:** a School of Chemistry and Life Science, Maoming University, Maoming 525000, People’s Republic of China

## Abstract

The title compound, C_16_H_23_NO_4_, is essentially planar except for the last two C atoms in each *n*-butyl group (r.m.s. deviation from the least-squares plane = 0.02 Å for 17 non-H atoms). In the crystal, inter­molecular N—H⋯O hydrogen bonds between the amine and carbonyl groups link the mol­ecules into one-dimensional chains.

## Related literature

For the related structure of 5-amino­isophthalic acid hemihydrate, see: Dobson & Gerkin (1998 ▶).
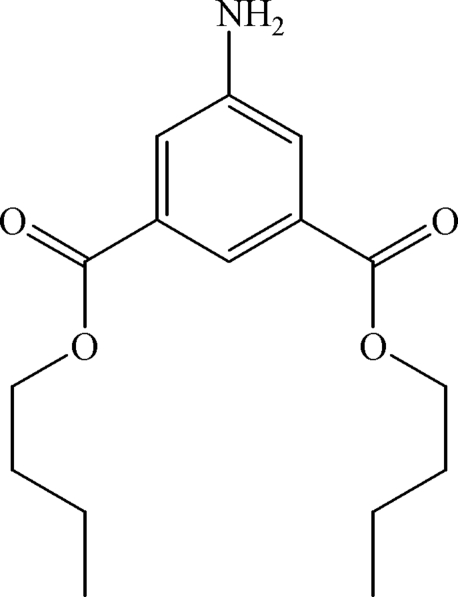


## Experimental

### 

#### Crystal data

C_16_H_23_NO_4_
*M* *_r_* = 293.35
Monoclinic, 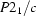

*a* = 9.4350 (19) Å
*b* = 9.1640 (18) Å
*c* = 20.166 (4) Å
β = 94.67 (3)°
*V* = 1737.8 (6) Å^3^
*Z* = 4
Mo *K*α radiation
μ = 0.08 mm^−1^
*T* = 296 K
0.12 × 0.10 × 0.08 mm

#### Data collection

Bruker APEXII CCD diffractometer
Absorption correction: multi-scan (*SADABS*; Bruker, 2001 ▶) *T*_min_ = 0.991, *T*_max_ = 0.994
8863 measured reflections
3169 independent reflections
1612 reflections with *I* > 2σ(*I*)
*R*_int_ = 0.029

#### Refinement

*R*[*F* ^2^ > 2σ(*F* ^2^)] = 0.062
*wR*(*F* ^2^) = 0.221
*S* = 1.04
3169 reflections
196 parameters
H atoms treated by a mixture of independent and constrained refinement
Δρ_max_ = 0.30 e Å^−3^
Δρ_min_ = −0.16 e Å^−3^

### 

Data collection: *APEX2* (Bruker, 2004 ▶); cell refinement: *SAINT-Plus* (Bruker, 2001 ▶); data reduction: *SAINT-Plus*; program(s) used to solve structure: *SHELXS97* (Sheldrick, 2008 ▶); program(s) used to refine structure: *SHELXL97* (Sheldrick, 2008 ▶); molecular graphics: *SHELXTL* (Sheldrick, 2008 ▶); software used to prepare material for publication: *SHELXTL*.

## Supplementary Material

Crystal structure: contains datablocks global, I. DOI: 10.1107/S1600536809024696/bi2379sup1.cif

Structure factors: contains datablocks I. DOI: 10.1107/S1600536809024696/bi2379Isup2.hkl

Additional supplementary materials:  crystallographic information; 3D view; checkCIF report

## Figures and Tables

**Table 1:** Hydrogen-bond geometry (Å, °)

*D*—H⋯*A*	*D*—H	H⋯*A*	*D*⋯*A*	*D*—H⋯*A*
N1—H1*A*⋯O1^i^	0.83 (5)	2.30 (6)	3.110 (4)	165 (6)
N1—H1*B*⋯O3^ii^	0.83 (6)	2.38 (6)	3.120 (4)	149 (5)

Symmetry codes: (i) 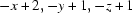; (ii) 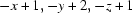.

**Fig. 1.**
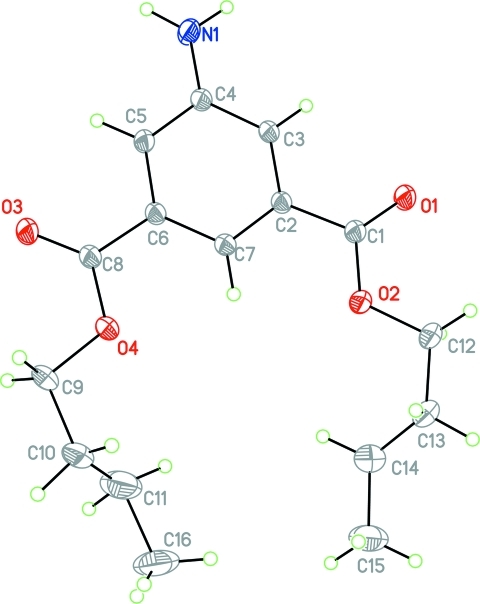
Molecular structure with 30% probability displacement ellipsoids for non-H atoms.

**Fig. 2.**
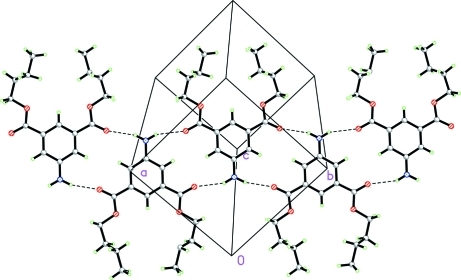
One-dimensional hydrogen-bonded motif.

## supplementary crystallographic information

### Comment

As a part of our ongoing research on the synthesis and structure of Schiff-base ligands based on 5-aminoisophthalic acid, we obtained the title compound from acid-catalysed esterification in 1-butanol.

### Experimental

5-Aminoisophthalic acid (10 g) was refluxed overnight in 1-butanol with catalysis of concentrated H_2_SO_4_. The solution was poured onto ice and adjusted to pH = 7, and the obtained powder was recrystallized from ethanol. Elemental analysis calculated: C 65.45, H 7.84, N 4.77%; found: C 65.42, H 7.81, N 4.70%.

### Refinement

All H atoms bound to C atoms were geometrically positioned and refined using a riding model, with C—H = 0.93 (aryl), 0.97 (methylene) or 0.96 Å (methyl), and with *U*_iso_(H) = *1.2*U_eq_(C) (aryl, methylene) or 1.5*U*_eq_(C) (methyl). H atoms on amino N were located from difference Fourier maps and their positions were refined freely, with *U*_iso_(H) = 1.5*U*_eq_(N). The refined N—H distances are 0.83 (5) and 0.83 (6) Å.

### Crystal data

**Table:**

C_16_H_23_NO_4_	*F*(000) = 632
*M**_r_* = 293.35	*D*_x_ = 1.121 Mg m^−^^3^
Monoclinic, *P*2_1_/*c*	Mo *K*α radiation, λ = 0.71073 Å
Hall symbol: -P 2ybc	Cell parameters from 3119 reflections
*a* = 9.4350 (19) Å	θ = 2.0–25.5°
*b* = 9.1640 (18) Å	µ = 0.08 mm^−^^1^
*c* = 20.166 (4) Å	*T* = 296 K
β = 94.67 (3)°	Block, colourless
*V* = 1737.8 (6) Å^3^	0.12 × 0.10 × 0.08 mm
*Z* = 4	

### Data collection

**Table:**

Bruker APEXII CCD diffractometer	3169 independent reflections
Radiation source: fine-focus sealed tube	1612 reflections with *I* > 2σ(*I*)
graphite	*R*_int_ = 0.029
φ and ω scans	θ_max_ = 25.5°, θ_min_ = 2.0°
Absorption correction: multi-scan (*SADABS*; Bruker, 2001)	*h* = −7→11
*T*_min_ = 0.991, *T*_max_ = 0.994	*k* = −11→10
8863 measured reflections	*l* = −24→23

### Refinement

**Table:**

Refinement on *F*^2^	Primary atom site location: structure-invariant direct methods
Least-squares matrix: full	Secondary atom site location: difference Fourier map
*R*[*F*^2^ > 2σ(*F*^2^)] = 0.062	Hydrogen site location: inferred from neighbouring sites
*wR*(*F*^2^) = 0.221	H atoms treated by a mixture of independent and constrained refinement
*S* = 1.04	*w* = 1/[σ^2^(*F*_o_^2^) + (0.1119*P*)^2^ + 0.1542*P*] where *P* = (*F*_o_^2^ + 2*F*_c_^2^)/3
3169 reflections	(Δ/σ)_max_ < 0.001
196 parameters	Δρ_max_ = 0.30 e Å^−^^3^
0 restraints	Δρ_min_ = −0.16 e Å^−^^3^

### Special details

**Table:**

Geometry. All esds (except the esd in the dihedral angle between two l.s. planes) are estimated using the full covariance matrix. The cell esds are taken into account individually in the estimation of esds in distances, angles and torsion angles; correlations between esds in cell parameters are only used when they are defined by crystal symmetry. An approximate (isotropic) treatment of cell esds is used for estimating esds involving l.s. planes.
Refinement. Refinement of F^2^ against ALL reflections. The weighted R-factor wR and goodness of fit S are based on F^2^, conventional R-factors R are based on F, with F set to zero for negative F^2^. The threshold expression of F^2^ > 2sigma(F^2^) is used only for calculating R-factors(gt) etc. and is not relevant to the choice of reflections for refinement. R-factors based on F^2^ are statistically about twice as large as those based on F, and R- factors based on ALL data will be even larger.

### Fractional atomic coordinates and isotropic or equivalent isotropic displacement parameters (Å^2^)

**Table:**

	*x*	*y*	*z*	*U*_iso_*/*U*_eq_	
O1	0.8685 (2)	0.3309 (2)	0.44186 (12)	0.1051 (8)	
O2	0.6509 (2)	0.3015 (2)	0.39278 (11)	0.0938 (7)	
O3	0.3317 (2)	0.8767 (3)	0.44890 (13)	0.1092 (8)	
O4	0.29283 (19)	0.6651 (2)	0.39827 (11)	0.0944 (7)	
N1	0.8560 (3)	0.8512 (4)	0.5269 (2)	0.1380 (15)	
H1A	0.933 (6)	0.815 (7)	0.540 (3)	0.207*	
H1B	0.831 (6)	0.926 (6)	0.546 (3)	0.207*	
C1	0.7518 (3)	0.3783 (3)	0.42708 (14)	0.0759 (8)	
C2	0.7015 (2)	0.5250 (3)	0.44442 (12)	0.0674 (7)	
C3	0.7986 (3)	0.6180 (3)	0.47724 (14)	0.0768 (8)	
H3A	0.8917	0.5868	0.4872	0.092*	
C4	0.7598 (3)	0.7572 (3)	0.49558 (15)	0.0856 (9)	
C5	0.6193 (3)	0.8002 (3)	0.47979 (15)	0.0808 (8)	
H5A	0.5905	0.8928	0.4919	0.097*	
C6	0.5223 (3)	0.7080 (3)	0.44666 (13)	0.0681 (7)	
C7	0.5629 (3)	0.5693 (3)	0.42860 (12)	0.0675 (7)	
H7A	0.4977	0.5068	0.4062	0.081*	
C8	0.3749 (3)	0.7609 (3)	0.43193 (15)	0.0784 (8)	
C9	0.1463 (3)	0.7056 (4)	0.38233 (19)	0.1024 (10)	
H9A	0.1406	0.7901	0.3533	0.123*	
H9B	0.1017	0.7296	0.4226	0.123*	
C10	0.0741 (4)	0.5806 (5)	0.3489 (3)	0.1371 (16)	
H10A	0.0657	0.5054	0.3822	0.165*	
H10B	−0.0218	0.6115	0.3344	0.165*	
C11	0.1293 (6)	0.5169 (9)	0.2960 (4)	0.220 (3)	
H11A	0.2257	0.4870	0.3101	0.265*	
H11B	0.1358	0.5912	0.2621	0.265*	
C12	0.0559 (7)	0.3906 (9)	0.2644 (5)	0.300 (6)	
H12A	0.1046	0.3602	0.2267	0.450*	
H12B	−0.0401	0.4170	0.2499	0.450*	
H12C	0.0553	0.3119	0.2958	0.450*	
C13	0.6881 (4)	0.1552 (3)	0.3734 (2)	0.1112 (11)	
H13A	0.7234	0.0992	0.4121	0.133*	
H13B	0.7614	0.1584	0.3424	0.133*	
C14	0.5572 (5)	0.0874 (4)	0.3415 (3)	0.1471 (16)	
H14A	0.5822	−0.0100	0.3277	0.177*	
H14B	0.4913	0.0762	0.3757	0.177*	
C15	0.4815 (6)	0.1559 (6)	0.2858 (3)	0.175 (2)	
H15A	0.5447	0.1639	0.2504	0.210*	
H15B	0.4570	0.2542	0.2986	0.210*	
C16	0.3471 (6)	0.0794 (7)	0.2586 (3)	0.205 (3)	
H16A	0.3044	0.1331	0.2212	0.307*	
H16B	0.2817	0.0740	0.2926	0.307*	
H16C	0.3698	−0.0173	0.2447	0.307*	

### Atomic displacement parameters (Å^2^)

**Table:**

	*U*^11^	*U*^22^	*U*^33^	*U*^12^	*U*^13^	*U*^23^
O1	0.0690 (13)	0.0819 (14)	0.160 (2)	0.0183 (10)	−0.0158 (13)	−0.0206 (13)
O2	0.0795 (13)	0.0724 (13)	0.1264 (16)	0.0090 (10)	−0.0112 (11)	−0.0208 (11)
O3	0.0665 (12)	0.0882 (15)	0.169 (2)	0.0181 (11)	−0.0143 (12)	−0.0307 (14)
O4	0.0608 (11)	0.0900 (14)	0.1280 (17)	0.0102 (10)	−0.0193 (10)	−0.0165 (12)
N1	0.0748 (18)	0.093 (2)	0.237 (4)	0.0125 (15)	−0.046 (2)	−0.060 (2)
C1	0.0668 (17)	0.0684 (17)	0.0917 (19)	0.0034 (14)	0.0007 (14)	−0.0048 (14)
C2	0.0591 (14)	0.0654 (15)	0.0775 (16)	0.0036 (12)	0.0031 (12)	0.0013 (12)
C3	0.0570 (14)	0.0715 (18)	0.0993 (19)	0.0082 (12)	−0.0090 (13)	−0.0062 (14)
C4	0.0631 (16)	0.0731 (19)	0.117 (2)	0.0039 (14)	−0.0137 (15)	−0.0129 (16)
C5	0.0621 (16)	0.0667 (16)	0.111 (2)	0.0084 (13)	−0.0059 (14)	−0.0116 (15)
C6	0.0563 (14)	0.0651 (16)	0.0821 (17)	0.0020 (12)	−0.0004 (12)	0.0021 (13)
C7	0.0604 (15)	0.0667 (16)	0.0747 (16)	0.0012 (12)	0.0006 (12)	0.0007 (12)
C8	0.0611 (15)	0.0721 (19)	0.100 (2)	0.0036 (14)	−0.0054 (14)	−0.0018 (15)
C9	0.0600 (17)	0.119 (3)	0.123 (2)	0.0091 (17)	−0.0191 (16)	−0.007 (2)
C10	0.080 (2)	0.156 (4)	0.168 (4)	0.000 (2)	−0.030 (2)	−0.036 (3)
C11	0.127 (4)	0.280 (8)	0.248 (7)	−0.001 (5)	−0.022 (4)	−0.138 (7)
C12	0.173 (6)	0.301 (10)	0.410 (13)	−0.007 (5)	−0.074 (6)	−0.230 (10)
C13	0.114 (3)	0.073 (2)	0.143 (3)	0.0126 (18)	−0.009 (2)	−0.0249 (19)
C14	0.155 (4)	0.088 (3)	0.190 (4)	0.007 (2)	−0.038 (3)	−0.042 (3)
C15	0.168 (4)	0.148 (4)	0.197 (5)	0.010 (3)	−0.052 (4)	−0.041 (4)
C16	0.140 (4)	0.228 (6)	0.235 (6)	−0.002 (4)	−0.053 (4)	−0.094 (5)

### Geometric parameters (Å, °)

**Table:**

O1—C1	1.198 (3)	C9—H9B	0.970
O2—C1	1.331 (3)	C10—C11	1.357 (6)
O2—C13	1.448 (4)	C10—H10A	0.970
O3—C8	1.197 (3)	C10—H10B	0.970
O4—C8	1.321 (3)	C11—C12	1.468 (8)
O4—C9	1.442 (3)	C11—H11A	0.970
N1—C4	1.368 (4)	C11—H11B	0.970
N1—H1A	0.83 (5)	C12—H12A	0.960
N1—H1B	0.83 (6)	C12—H12B	0.960
C1—C2	1.478 (4)	C12—H12C	0.960
C2—C3	1.380 (4)	C13—C14	1.481 (5)
C2—C7	1.382 (3)	C13—H13A	0.970
C3—C4	1.385 (4)	C13—H13B	0.970
C3—H3A	0.930	C14—C15	1.426 (7)
C4—C5	1.395 (4)	C14—H14A	0.970
C5—C6	1.377 (4)	C14—H14B	0.970
C5—H5A	0.930	C15—C16	1.513 (7)
C6—C7	1.385 (3)	C15—H15A	0.970
C6—C8	1.480 (4)	C15—H15B	0.970
C7—H7A	0.930	C16—H16A	0.960
C9—C10	1.468 (5)	C16—H16B	0.960
C9—H9A	0.970	C16—H16C	0.960
			
C1—O2—C13	116.8 (2)	C9—C10—H10B	107.3
C8—O4—C9	117.0 (2)	H10A—C10—H10B	106.9
C4—N1—H1A	116 (4)	C10—C11—C12	118.9 (7)
C4—N1—H1B	122 (4)	C10—C11—H11A	107.6
H1A—N1—H1B	117 (5)	C12—C11—H11A	107.6
O1—C1—O2	122.8 (2)	C10—C11—H11B	107.6
O1—C1—C2	125.3 (3)	C12—C11—H11B	107.6
O2—C1—C2	112.0 (2)	H11A—C11—H11B	107.0
C3—C2—C7	120.6 (2)	C11—C12—H12A	109.5
C3—C2—C1	117.5 (2)	C11—C12—H12B	109.5
C7—C2—C1	121.9 (2)	H12A—C12—H12B	109.5
C2—C3—C4	121.1 (2)	C11—C12—H12C	109.5
C2—C3—H3A	119.4	H12A—C12—H12C	109.5
C4—C3—H3A	119.4	H12B—C12—H12C	109.5
N1—C4—C3	121.5 (2)	O2—C13—C14	107.1 (3)
N1—C4—C5	120.7 (3)	O2—C13—H13A	110.3
C3—C4—C5	117.8 (2)	C14—C13—H13A	110.3
C6—C5—C4	121.3 (3)	O2—C13—H13B	110.3
C6—C5—H5A	119.4	C14—C13—H13B	110.3
C4—C5—H5A	119.4	H13A—C13—H13B	108.6
C5—C6—C7	120.2 (2)	C15—C14—C13	120.2 (5)
C5—C6—C8	118.2 (2)	C15—C14—H14A	107.3
C7—C6—C8	121.5 (2)	C13—C14—H14A	107.3
C2—C7—C6	119.0 (2)	C15—C14—H14B	107.3
C2—C7—H7A	120.5	C13—C14—H14B	107.3
C6—C7—H7A	120.5	H14A—C14—H14B	106.9
O3—C8—O4	122.5 (2)	C14—C15—C16	115.7 (5)
O3—C8—C6	124.7 (3)	C14—C15—H15A	108.4
O4—C8—C6	112.8 (3)	C16—C15—H15A	108.4
O4—C9—C10	107.6 (3)	C14—C15—H15B	108.4
O4—C9—H9A	110.2	C16—C15—H15B	108.4
C10—C9—H9A	110.2	H15A—C15—H15B	107.4
O4—C9—H9B	110.2	C15—C16—H16A	109.5
C10—C9—H9B	110.2	C15—C16—H16B	109.5
H9A—C9—H9B	108.5	H16A—C16—H16B	109.5
C11—C10—C9	120.2 (4)	C15—C16—H16C	109.5
C11—C10—H10A	107.3	H16A—C16—H16C	109.5
C9—C10—H10A	107.3	H16B—C16—H16C	109.5
C11—C10—H10B	107.3		
			
C13—O2—C1—O1	−0.1 (5)	C1—C2—C7—C6	179.6 (2)
C13—O2—C1—C2	−179.6 (3)	C5—C6—C7—C2	0.1 (4)
O1—C1—C2—C3	3.6 (4)	C8—C6—C7—C2	−179.1 (2)
O2—C1—C2—C3	−176.9 (2)	C9—O4—C8—O3	0.0 (4)
O1—C1—C2—C7	−176.5 (3)	C9—O4—C8—C6	179.1 (2)
O2—C1—C2—C7	3.0 (4)	C5—C6—C8—O3	−2.9 (5)
C7—C2—C3—C4	0.5 (4)	C7—C6—C8—O3	176.4 (3)
C1—C2—C3—C4	−179.6 (3)	C5—C6—C8—O4	178.0 (2)
C2—C3—C4—N1	−178.7 (3)	C7—C6—C8—O4	−2.7 (4)
C2—C3—C4—C5	0.0 (4)	C8—O4—C9—C10	−176.6 (3)
N1—C4—C5—C6	178.2 (3)	O4—C9—C10—C11	−51.7 (6)
C3—C4—C5—C6	−0.4 (5)	C9—C10—C11—C12	179.0 (7)
C4—C5—C6—C7	0.4 (4)	C1—O2—C13—C14	175.1 (3)
C4—C5—C6—C8	179.6 (3)	O2—C13—C14—C15	56.2 (6)
C3—C2—C7—C6	−0.5 (4)	C13—C14—C15—C16	−178.3 (4)

### Hydrogen-bond geometry (Å, °)

**Table:**

*D*—H···*A*	*D*—H	H···*A*	*D*···*A*	*D*—H···*A*
N1—H1A···O1^i^	0.83 (5)	2.30 (6)	3.110 (4)	165 (6)
N1—H1B···O3^ii^	0.83 (6)	2.38 (6)	3.120 (4)	149 (5)

Symmetry codes: (i) −*x*+2, −*y*+1, −*z*+1; (ii) −*x*+1, −*y*+2, −*z*+1.
